# Novel Small-Molecule Hybrid-Antibacterial Agents against *S. aureus* and MRSA Strains

**DOI:** 10.3390/molecules27010061

**Published:** 2021-12-23

**Authors:** Robin Gehrmann, Tobias Hertlein, Elisa Hopke, Knut Ohlsen, Michael Lalk, Andreas Hilgeroth

**Affiliations:** 1 Institute of Pharmacy, Martin Luther University Halle-Wittenberg, 06120 Halle, Germany; robin.gehrmann@pharmazie.uni-halle.de; 2 Institute of Molecular Infection Biology, Julius Maximilians University Wuerzburg, 97080 Wuerzburg, Germany; tobias.hertlein@uni-wuerzburg.de (T.H.); elisa.hopke@stud-mail.uni-wuerzburg.de (E.H.); knut.ohlsen@uni-wuerzburg.de (K.O.); 3 Institute of Biochemistry, Ernst Moritz Arndt University Greifswald, 17489 Greifswald, Germany; lalk@uni-greifswald.de

**Keywords:** antibacterial activity, synthesis, substituent, structure–activity, inhibition

## Abstract

Ongoing resistance developments against antibiotics that also affect last-resort antibiotics require novel antibacterial compounds. Strategies to discover such novel structures have been dimerization or hybridization of known antibacterial agents. We found novel antibacterial agents by dimerization of indols and hybridization with carbazoles. They were obtained in a simple one-pot reaction as bisindole tetrahydrocarbazoles. Further oxidation led to bisindole carbazoles with varied substitutions of both the indole and the carbazole scaffold. Both the tetrahydrocarbazoles and the carbazoles have been evaluated in various *S. aureus* strains, including MRSA strains. Those 5-cyano substituted derivatives showed best activities as determined by MIC values. The tetrahydrocarbazoles partly exceed the activity of the carbazole compounds and thus the activity of the used standard antibiotics. Thus, promising lead compounds could be identified for further studies.

## 1. Introduction

Antibiotic resistance poses a threat to human health today [1]. It has been predicted that without urgent actions in 2050 10 million people a year will die from bacterial infections that cannot be treated due to antibiotic resistance [2,3]. Since the discovery of penicillin, numerous antibacterial drugs have been discovered and developed to classes of antibiotics with various antibacterial effects [1,2,4]. The development of resistance to individual antibiotics was not a problem for the therapy, as the number of alternatives was sufficient [2,5]. However, in the case of resistance to various antibiotics, treatment with antibiotics of last-resort remained an option [1,6]. In the meantime, it has to be stated that resistances have also been described against these antibiotics of last-resort [6,7]. New antibiotics, such as linezolid or daptomycin, were discovered up to the end of the last century, but as of today there is a lack of novel compounds [1,8].

The reason for this is the fact that the pharmaceutical companies no longer invest in the development of novel antibiotics because the costs of development are too high with respect to the benefits [2,8]. Antibiotics which must be available to all rich and poor countries should have low prices and, moreover, in the case of the development of a new drug with a novel mode of action, innovation will be restricted in use as a potential antibiotic of last-resort and thus the benefit for the pharmaceutical companies will also be limited [2,8,9,10].

In consequence, novel drugs are needed. However, most of the described antibacterial compounds are natural products produced by bacteria, fungi or plants with complex structures [11,12]. These isolated novel compounds are difficult to synthesize and thus production costs are also estimated to be high [13]. In contrast, the number of synthetic antibiotics is very low and new developments mostly did not come to the clinic [2,14].

Both the misuse and overuse of antibiotics and the ability of bacteria to transfer resistances also between different species in so-called horizontal transfer accelerated the development of the current crisis [1,15,16]. One option to discover new potential antibiotic drugs is the dimerization or hybridization of known antibacterial structure elements [17,18]. Applying this strategy, enhanced antibacterial activities have been described for bisindole compounds compared to respective single indole derivatives [19,20]. Structurally, those bisindoles consist of single indole derivatives that have been linked by an alkyl chain with a partly aromatic core [21,22]. Another strategy has been the hybridization of indole with other antibacterial structure elements like imidazoles, quinolones or sulphonamides [23,24,25].

We synthesized novel bisindole hybrid molecules and thus have been able to combine both techniques. The research design was based on the chemical knowledge that indole reacts with a monoaldehyde to give bisindolyl methane derivatives with various biological activities, including antibacterial properties that depend on the substitution patterns [26,27]. We used a bisaldehyde to form a bisindolyl methane by the reaction of one aldehyde function and expected a reaction of the second aldehyde function with a third indole compound in consequence. The resulting carbenium function under the chosen acid conditions underwent ring closure. In this way, novel tetrahydrocarbazoles were formed and carbazoles were obtained by oxidation of the tetrahydrocarbazoles that were substituted with two indole residues. For carbazoles alone, moderate to low antibacterial activities have been described against *Staphylococcus (S.*) *aureus* strains [28,29]. Recently described bisindole compounds also show antibacterial activities towards *S. aureus* and methicillin-resistant MRSA strains [30]. Therefore, we decided to evaluate our novel compounds with various indole substitutions as antibacterial agents against *S. aureus* strains. We discovered first promising lead structures of the various synthesized compound classes. 

## 2. Results and Discussion

### 2.1. Formation of the Bisindole Tetrahydrocarbazoles and the Carbazoles

The bisindole tetrahydrocarbazoles were obtained by the reaction of indole **1** and succinaldehyde **2** in acetic acid under mild conditions at room temperature. The succinaldehyde was freshly prepared from 2,5-dimethoxyfurane by a hydrochloric acid treatment under heating at 60 °C [31].

**Scheme 1:**
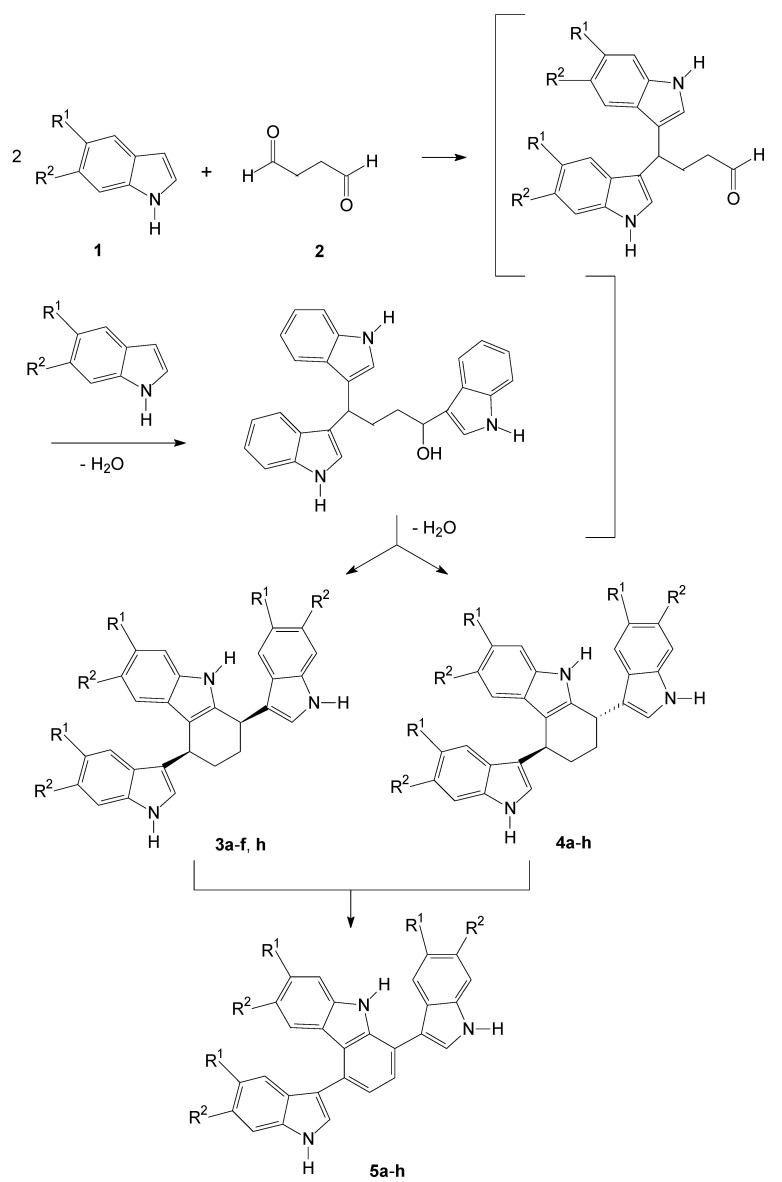
Formation of Target Compounds.

As indole and a monoaldehyde are known to given bisindolyl methane derivatives [30,31], it is suggested that one of the aldehyde functions of the succinaldehyde first reacted with two indole compounds at the electrophilic 3-position of each indole, similar to the reaction of those bisindolyl methane compounds (Scheme 1). Then, the second aldehyde function reacted with the third indole at the corresponding 3-position. The final ring closure to the tetrahydro cyclohexene partial structure of the tetrahydrocarbazole scaffold has been achieved by reaction of the resulting carbenium function after water elimination of the former aldehyde with the 2-position of the third indole compound.

In the tetrahydrocarbazole compounds **3a**–**f** and **h**, the attached indole residues have a *cis* orientation of the indole residues, whereas the orientation in compounds **4a**–**h** is *trans*. Due to the hindered rotability of the *cis* positioned indole residues, there is an anisotropic effect of the aromatic indole residue that explained the observed high field shift of the corresponding methin proton signal in the ^1^H NMR spectra, so that the two methin proton signals of the tetrahydro partial structure appear closer together in the *cis* compounds compared to their appearance in the spectra of *trans* compounds **4a**–**h** where they are found with a higher distance due the influence of the indole nitrogen neighbourhood on one methin proton.

The final bisindole carbazoles **5a**–**h** were obtained by the treatment of the mixed tetrahydrocarbazole compounds **3a**–**f** and **h** and **4a**–**h** with an excess of oxidizing 2,3-dichloro-5,6-dicyano-*p*-benzoquinone in methanol. In the ^1^H NMR spectra, the aliphatic proton signals of the tetrahydro partial structure disappeared due to the aromatization. Moreover, that reaction additionally proved the discussed structures of both tetrahydro intermediate compounds **3a**–**f** and **h** and **4a**–**h**.

### 2.2. Antibacterial Activity of the Bisindole Tetrahaydrocarbazoles and the Carbazoles

The antibacterial compound activity was determined as the minimal inhibitory concentration (MIC) of the bacterial growth using the two-fold serial dilution technique. Stock solutions of the compounds in DMSO were prepared and two-fold serial dilutions were applied. Oxacillin and ciprofloxacin have been used as standards. All compounds have been initially tested against one MRSA strain (USA300 Lac* lux) and in the case of an activity at a concentration <16 µg/mL, that meant an activity better than that of the used standard antibiotics, the activities against another MRSA strain, JE2, and two methicillin-sensitive *S. aureus* (MSSA) strains, ATCC6538 and HG003, were determined. In the MRSA strain JE2 plasmids encoded resistance genes have been deleted.

We started with the MIC value determination for the bisindole carbazole hybride compounds **5a**–**h**. The values are shown in Table 1.

For compound **5a** with the unsubstituted indole residues the MIC value in the USA300 Lac* lux strain was determined with 8 µg/mL. That meant a fourfold better activity than that of the used standards. The activities towards the other MRSA strain and the MSSA strain ATCC6538 was found increased with MIC values of 4 and 2 µg/mL. When a chloro substitution was introduced in the 5-position of the indole in compound **5b** the activity towards USA300 Lac* lux was similar as for **5a**. However, activity towards the MSSA strains was different, against ATCC6538 more active and against HG003 less active. When the 5-chloro substitution moved to the 6-position of the indole residues in derivative **5c**, the MRSA activity was found reduced in USA300 Lac* lux. A bromo function in the 5-position of the indole residues of compound **5d** mainly lowered the activity to a value of just 64 µg/mL in USA300 Lac* lux. Placed in the 6-position of the indoles in derivative **5e** the compound was found no longer active with a MIC value >128 µg/mL. A benzyloxy function in both the 5- and the 6-position of the indole residues of compounds **5f** and **5g** were unfavourable with MIC values of 128 µg/mL and >128 µg/mL, respectively. Finally, a cyano substituent was introduced in the 5-indole position of compound **5h**. The activities towards all the MRSA and the MSSA strains were similar than that of compound **5b** except that towards ATCC6538 that was found slightly lowered. It can be stated that a 5-substitution of the indole is more favourable than a respective 6-substitution with best activities for a 5-chloro and a 5-cyano substitution.

Next, we evaluated our *trans* indole substituted compound series **4**-**h** in order to investigate the importance of the aromatized carbazole scaffold for the observed antibacterial activity. Compound **4a** with the unsubstituted indole residues showed an improved activity towards the MRSA strain USA300 Lac* lux and towards ATCC6538 compared to the corresponding carbazole compound **5a**. The activity towards the MSSA strain HG003 was found decreased. The 5-chloro indole compound **4b** showed similar and improved activities towards the MRSA strains as **5b**, whereas the activity towards the MSSA strains was decreased. The activities of the 6-chloro and the 5-bromo compounds **4c** and **4d** were similar with a MIC of 16 µg/mL comparable to those of the corresponding carbazole compound **5c**. If compared to derivative **5d** the activity of compound **4d** was found increased. Similar to compound **5e** derivative **4e** with the 6-bromo indole substitution showed no more antibacterial activity. Moreover, both benzyloxy indole substituted compounds **4f** and **4g** were obviously no longer active similar to the carbazole compounds **5f** and **5g**. Finally, the 5-cyano indole compound **4h** showed a partly improved activity towards the MRSA strains compared to the carbazole derivative **5h** and an increased MSSA activity towards ATCC6538. Generally, it can be stated that activity of the *trans* indole compound series was similar to that of the carbazole series with partial improvements towards the MRSA strains and decreases towards the MSSA strains.

The *cis* indol substituted compound **3a** from the second tetrahydrocarbazole series showed mainly improved activities towards the MRSA strains with MIC values about 1–2 µg/mL and also against the MSSA strains with an activity that partly exceeds that of the used standard ciprofloxacin.

The 5-chloro indole substitution in derivative **3b** was as favourable as in compound **4b** with similar results towards the MRSA strains and again an improved activity towards the MSSA strain HG003.

The 6-chloro indole and the 5-bromo indole substitution in derivatives **3c** and **3d** lowered the MRSA activity similar to compounds **4c** and **4d** of the *trans* indole series. Similar to compounds **4e** and **4f** the 6-bromo and the 5-benzyloxy function in derivatives **3e** and **3f** led to a loss of activity with MIC values >128 µg/mL.

Finally, the 5-cyano substitution in derivative **3h** led to improved activities towards the MRSA strains and the MSSA strain HG003 compared to the derivative **4h**.

In consequence, the *cis* indole substitution was more favourable in the most active compounds than the *trans* substitution towards MRSA and MSSA strains.

## 3. Materials and Methods

### 3.1. Chemical Reagents and Instruments

Commercial reagents were used without further purification. The ^1^H-NMR spectra (400 MHz) were measured using tetramethylsilane as an internal standard. Thin layer chromatography (TLC) was performed on E. Merck 5554 silica gel plates. The mass spectra were recorded on a Finnigan LCQ Classic mass spectrometer.

### 3.2. General Procedure for the Synthesis of Bisindolyl Tetrahydrocarbazoles ***3*** and ***4***

2 mmol of the freshly distilled succinaldehyde **2** was dissolved in 15 mL acetic acid (100%). After addition of 5 mmol of the indole derivative, the solution color changed. The mixture was stirred for at least 24 h at room temperature protected from light. The reaction proceeding was followed by TLC, indicating the formation of two products. When no more changes in the detectable product formation were observed within the mixture according to TLC, the work-up procedure started with adjusting the pH-value to 6–7 using sodium hydroxide solution (10%) under precipitate formation. The precipitate was filtered and washed with distilled water. Then it was dissolved in ethyl acetate and dried over magnesium sulfate. After filtration, the solvent was removed in vacuum. The oily residue was separated by column chromatography over silica gel to result in eluated fractions containing each compounds **3** and **4**, respectively.

(1*R*,4*S*)-1,4-Di(1*H*-indol-3-yl)-2,3,4,9-tetrahydro-1*H*-carbazole (**3a**). Yield 21%, light pink powder; mp 167–168 °C; ^1^H NMR (DMSO-d_6_) δ = 10.89 (d, *^3^J_N′/2′_* = 2.4 Hz, 1H, N′-H), 10.69 (d, *^3^J_N″/2″_* = 2.3 Hz, 1H, N″-H), 10.45 (s, 1H, N-H), 7.61 (d_br_, *^3^J_4″/5″_* = 7.9 Hz, *^4^J_4″/6″_*, 1H, 4″-H), 7.39 (d_br_, *^3^J_4′/5′_* = 7.9 Hz, *^4^J_4′/6′_*, 1H, 4′-H), 7.36 (dd, *^3^J_7′/6′_* = 8.2 Hz, *^4^J_7′/5′_* = 0.9 Hz, 1H, 7′-H), 7.33 (dd, *^3^J_7″/6″_* = 8.2 Hz, *^4^J_7″/5″_* = 0.9 Hz, 1H, 7″-H), 7.18 (dd, *^3^J_8/7_* = 7.9 Hz, *^4^J_8/6_* = 0.9 Hz, 1H, 8-H), 7.08 (d, *^3^J_2′/N′_* = 2.4 Hz, 1H, 2′-H), 7.04 (ddd, *^3^J_6′/7″; 6″/7″_* = 7.9 Hz, *^3^J_6′/5′; 6″/5″_* = 7.4 Hz, *^4^J_6′/4′; 6″/4″_* = 0.9 Hz, 2H: 6″-H; 6′-H), 6.94 (ddd, *^3^J_5″/4″_* = 7.9 Hz, *^3^J_5″/6″_* = 7.4 Hz, *^4^J_5″/7″_* = 0.9 Hz, 1H, 5″-H), 6.90 (ddd, *^3^J_5′/4′_* = 7.9 Hz, *^3^J_5′/6′_* = 7.4 Hz, *^4^J_5′/7′_* = 0.9 Hz, 1H, 5′-H), 6.89 (dd, *^3^J_5/6_* = 8.2 Hz, *^4^J_5/7_* = 1.2 Hz, 1H, 5-H), 6.87 (ddd, *^3^J_7/8_* = 7.9 Hz, *^3^J_7/6_* = 7.4 Hz, *^4^J_7/5_* = 1.2 Hz, 1H, 7-H), 6.81 (d, *^3^J_2″/N″_* = 2.3 Hz, 1H, 2″-H), 6.70 (ddd, *^3^J_6/5_* = 8.2 Hz, *^3^J_6/7_* = 7.4 Hz, *^4^J_6/8_* = 0.9 Hz, 1H, 6-H), 4.55 (t, *^3^J_4/3,3_* = 5.3 Hz, 1H, 4-H), 4.50 (t, *^3^J_1/2,2_* = 6.4 Hz, 1H, 1-H), 2.17–2.09 (m, 2H, 3,3-H), 2.08–2.02 (m, 2H, 2,2-H); *m*/*z* (ESI) 400.37 (100, [M−H]^−^).

(1*R*,4*S*)-6-Chloro-1,4-bis(5-chloro-1*H*-indol-3-yl)-2,3,4,9-tetrahydro-1*H*-carbazole (**3b**). Yield 16%, light beige powder; mp 149–152 °C; ^1^H NMR (DMSO-d_6_) δ = 11.18 (d, *^3^J_N′/2′_* = 2.3 Hz, 1H, N′-H), 11.03 (d, *^3^J_N″/2″_* = 2.4 Hz, 1H, N″-H), 10.77 (s, 1H, N-H), 7.61 (d, *^4^J_4″/6″_* = 2.1 Hz, 1H, 4″-H), 7.44 (d, *^4^J_4′/6′_* = 2.1 Hz, 1H, 4′-H), 7.40 (d, *^3^J_7′/6′_* = 8.5 Hz, 1H, 7′-H), 7.38 (d, *^3^J_7″/6″_* = 8.5 Hz, 1H, 7″-H), 7.23 (d, *^3^J_8/7_* = 8.5 Hz, 1H, 8-H), 7.17 (d, *^3^J_2′/N′_* = 2.3 Hz, 1H, 2′-H), 7.07 (dd, *^3^J_6′/7′_* = 8.5 Hz, *^4^J_6′/4′_* = 2.1 Hz, 1H, 6′-H), 7.07 (dd, *^3^J_6″/7″_* = 8.5 Hz, *^4^J_6″/4″_* = 2.1 Hz, 1H, 6″-H), 6.93 (dd, *^3^J_7/8_* = 8.5 Hz, *^4^J_7/5_* = 2.1 Hz, 1H, 7-H), 6.92 (d, *^3^J_2″/N″_* = 2.4 Hz, 1H, 2″-H), 6.86 (d, *^4^J_5/7_* = 2.1 Hz, 1H, 5-H), 4.55 (t, *^3^J_4/3,3_* = 5.1 Hz, 1H, 4-H), 4.52 (t, *^3^J_1/2,2_* = 5.1 Hz, 1H, 1-H), 2.23–2.12 (m, 2H, 3,3-H), 2.05–1.93 (m, 2H, 2,2-H); *m*/*z* (ESI) 502.17 (100, [M−H]^−^); 504.19 (86, [M−H]^−^); 506.17 (29, [M−H]^−^).

(1*R*,4*S*)-7-Chloro-1,4-bis(6-chloro-1*H*-indol-3-yl)-2,3,4,9-tetrahydro-1*H*-carbazole (**3c**). Yield 19%, beige powder; mp 172–173 °C; ^1^H NMR (DMSO-d_6_) δ = 11.07 (d, *^3^J_N′/2′_* = 2.3 Hz, 1H, N′-H), 10.89 (d, *^3^J_N″/2″_* = 2. Hz, 1H, N″-H), 10.55 (s, 1H, N-H), 7.41 (d, *^4^J_7′/5′_* = 2.0 Hz, 1H, 7′-H), 7.37 (d, *^4^J_7″/5″_* = 2.2 Hz, 1H, 7″-H), 7.33 (d, *^3^J_4″/5″_* = 8.5 Hz, 1H, 4″-H), 7.22 (d, *^3^J_4′/5′_* = 8.5 Hz, 1H, 4′-H), 7.14 (d, *^4^J_8/6_* = 1.9 Hz, 1H, 8-H), 7.13 (d, *^4^J_2′/N′_* = 2.4 Hz, 1H, 2′-H), 7.01 (d, *^4^J_2″/N″_* = 2.3 Hz, 1H, 2″-H), 6.90 (dd, *^3^J_5′/4′_* = 8.5 Hz, *^4^J_5′/7′_* = 1.9 Hz, 1H, 5′-H), 6.85 (dd, *^3^J_5″/4″_* = 8.5 Hz, *^4^J_5″/7″_* = 2.0 Hz, 1H, 5″-H), 6.69 (d, *^3^J_5/6_* = 8.5 Hz, 1H, 5-H), 6.65 (dd, *^3^J_6/5_* = 8.5 Hz, *^4^J_6/8_* = 1.9 Hz, 1H, 6-H), 4.58 (t, *J_4/3,3_* = 5.3 Hz, 1H, 4-H), 4.55 (t, *J_1/2,2_* = 5.4 Hz, 1H, 1-H), 2.23–2.17 (m, 2H, 3,3-H), 2.04–1.95 (m, 2H, 2,2-H); *m*/*z* (ESI) 502.23 (100, [M−H]^−^); 504.21 (85, [M−H]^−^).

(1*R*,4*S*)-6-Bromo-1,4-bis(5-bromo-1*H*-indol-3-yl)-2,3,4,9-tetrahydro-1*H*-carbazole (**3d**). Yield 24%, light beige powder; mp 146–148 °C; ^1^H NMR (DMSO-d_6_) δ = 11.19 (d, *^3^J_N′/2′_* = 2.3 Hz, 1H, N′-H), 11.05 (d, *^3^J_N″/2″_* = 2.4 Hz, 1H, N″-H), 10.77 (s, 1H, N-H), 7.76 (d, *^4^J_4″/6″_* = 2.0 Hz, 1H, 4″-H), 7.57 (d, *^4^J_4′/6′_* = 1.9 Hz, 1H, 4′-H), 7.36 (d, *^3^J_7″/6″_* = 8.6 Hz, 1H, 7″-H), 7.34 (d, *^3^J_7′/6′_* = 8.6 Hz, 1H, 7′-H), 7.19 (d, *^3^J_8/7′_* = 8.6 Hz, 1H, 8-H), 7.18 (dd, *^3^J_6″/7″_* = 8.6 Hz, *^4^J_6″/4″_* = 2.0 Hz, 1H, 6″-H), 7.17(dd, *^3^J_6′/7′_* = 8.6 Hz, *^4^J_6′/4′_* = 1.9 Hz, 1H, 6′-H), 7.16 (d, *^3^J_2′/N′_* = 2.3 Hz, 1H, 2′-H), 7.04 (dd, *^3^J_7/8_* = 8.6 Hz, *^4^J_7/5′_* = 2.0 Hz, 1H, 7-H), 7.01 (d, *^4^J_5/7_* = 2.0 Hz, 1H, 5-H), 6.88 (d, *^3^J_2″/N″_* = 2.4 Hz, 1H, 2″-H), 4.55 (t, *J_1/2,2_* = 5.2 Hz, 1H, 1-H), 4.51 (t, *J_4/3,3_* = 5.2 Hz, 1H, 4-H), 2.18–2.07 (m, 2H, 3,3-H), 2.07–1.94 (m, 2H, 2,2-H); *m*/*z* (ESI) 636.12 (100, [M−H]^−^); 638.11 (92, [M−H]^−^); 640.11 (34, [M−H]^−^); 634.18 (30, [M−H]^−^).

(1*R*,4*S*)-7-Bromo-1,4-bis(5-bromo-1*H*-indol-3-yl)-2,3,4,9-tetrahydro-1*H*-carbazole (**3e**). Yield 12%, light pink powder; mp 151–153 °C; ^1^H NMR (DMSO-d_6_) δ = 11.08 (d, *^3^J_N′/2′_* = 2.4 Hz, 1H, N′-H), 10.88 (d, *^3^J_N″/2″_* = 2.4 Hz, 1H, N″-H), 10.71 (s, 1H, N-H), 7.57 (d, *^4^J_7′/5′_* = 1.8 Hz, 1H, 7′-H), 7.53 (d, *^4^J_7″/5″_* = 1.8 Hz, 1H, 7″-H), 7.52 (d, *^3^J_4″/5″_* = 8.3 Hz, 1H, 4″-H), 7.36 (d, *^4^J_8/6_* = 1.8 Hz, 1H, 8-H), 7.33 (d, *^3^J_4′/5′_* = 8.3 Hz, 1H, 4′-H), 7.13 (d, *^3^J_2′/N′_* = 2.4 Hz, 1H, 2′-H), 7.07 (dd, *^3^J_5″/4″_* = 8.3 Hz, *^4^J_5″/7″_* = 1.8 Hz, 1H, 5″-H), 7.07 (dd, *^3^J_5′/4′_* = 8.3 Hz, *^4^J_5′/7′_* = 1.8 Hz, 1H, 5′-H), 6.88 (d, *^3^J_2″/N″_* = 2.4 Hz, 1H, 2″-H), 6.86 (dd, *^3^J_6/5_* = 8.4 Hz, *^4^J_6/8_* = 1.8 Hz, 1H, 6-H), 6.80 (d, *^3^J_5/6_* = 8.4 Hz, 1H, 5-H), 4.54 (t, *^3^J_1/2,2_* = 5.5 Hz, 1H, 1-H), 4.51 (t, *^3^J_4/3,3_* = 5.5 Hz, 1H, 4-H), 2.25–2.18 (m, 2H, 3,3-H), 2.03–1.93 (m, 2H, 2,2-H); *m*/*z* (ESI) 638.09 (100, [M−H]^−^); 636.13 (93, [M−H]^−^); 634.17 (39, [M−H]^−^); 640.06 (34, [M−H]^−^).

(1*R*,4*S*)-6-(Benzyloxy)-1,4-bis(5-(benzyloxy)-1*H*-indol-3-yl)-2,3,4,9-tetrahydro-1*H*-carbazole (**3f**). Yield 22%, red brownish powder; mp 89–91 °C; ^1^H NMR (DMSO-d_6_) δ = 10.71 (d, *^3^J_N′/2′_* = 2.3 Hz, 1H, N′-H), 10.56 (d, *^3^J_N″/2″_* = 2.4 Hz, 1H, N″-H), 10.26 (s, 1H, N-H), 7.40 (dd, *^3^J_2,6-Ph′/3,5-Ph′_* = 7.9 Hz, *^4^J_2,6-Ph′/4-Ph′_* = 1.1 Hz, 2H, 2,6-Ph′-H), 7.40–7.20 (m, 13H, Ar-H), 7.25 (d, *^3^J_7″/6″_* = 8.6 Hz, 1H, 7″-H), 7.24 (d, *^3^J_7′/6′_* = 8.6 Hz, 1H, 7′-H), 7.08 (d, *^3^J_8/7_* = 8.6 Hz, 1H, 8-H), 7.06 (d, *^4^J_4′/6′_* = 2.3 Hz, 1H, 4′-H), 6.94 (d, *^3^J_2′/N′_* = 2.3 Hz, 1H, 2′-H), 6.81 (d, *^3^J_2″/N″_* = 2.4 Hz, 1H, 2″-H), 6.79 (dd, *^3^J_6″/7″_* = 8.6 Hz, *^4^J_6″/4″_* = 2.4 Hz, 1H, 6″-H), 6.77 (dd, *^3^J_6′/7′_* = 8.6 Hz, *^4^J_6′/4′_* = 2.3 Hz, 1H, 6′-H), 6.75 (d, *^4^J_4″/6″_* = 2.4 Hz, 1H, 4″-H), 6.61 (dd, *^3^J_7/8_* = 8.6 Hz, *^4^J_7/5_* = 2.5 Hz, 1H, 7-H), 6.47 (d, *^4^J_5/7_* = 2.5 Hz, 1H, 5-H), 5.01 (s, 2H, O-CH_2_-Ph′), 4.84 (s, 2H, O-CH_2_-Ph″), 4.76–4.66 (m, 2H, O-CH_2_-Ph), 4.52–4.45 (m, 2H, 1-H; 4-H), 2.20–2.10 (m, 2H, 3,3-H), 2.01–1.81 (m, 2H, 2,2-H); *m*/*z* (ESI) 742.33 (100, [M+Na]^+^); 720.26 (13, [M+H]^+^).

3,3′-((1*R*,4*S*)-6-Cyano-2,3,4,9-tetrahydro-1*H*-carbazole-1,4-diyl)bis(1*H*-indole-5-carbonitrile) (**3h**). Yield 8%, light pink powder; mp 184–186 °C; ^1^H NMR (DMSO-d_6_) δ = 11.60 (d, *^3^J_N′/2_* = 2.4 Hz, 1H, N′-H), 11.44 (d, *^3^J_N″/2″_* = 2.4 Hz, 1H, N″-H), 11.15 (s, 1H, N-H), 8.14 (d, *^4^J_4″/6″_* = 1.5 Hz, 1H, 4″-H), 8.04 (d, ^4^*J_4′/6′_* = 1.5 Hz, 1H, 4′-H), 7.56 (d, *^3^J_7′/6′_* = 8.5 Hz, 1H, 7′-H), 7.54 (d, *^3^J_7″/6″_* = 8.6 Hz, 1H, 7″-H), 7.44 (dd, *^3^J_6′/7′_* = 8.5 Hz, *^4^J_6′/4′_* = 1.5 Hz, 1H, 6′-H), 7.43 (dd, *^3^J_6″/7″_* = 8.6 Hz, *^4^J_6″/4″_* = 1.5 Hz, 1H, 6″-H), 7.39 (d, *^3^J_8/7_* = 8.5 Hz, 1H, 8-H), 7.31 (dd, *^3^J_7/8_* = 8.5 Hz, *^4^J_7/5_* = 1.6 Hz, 1H, 7-H), 7.30 (d, *^3^J_2′/N′_* = 2.4 Hz, 1H, 2′-H), 7.29 (d, *^4^J_5/7_* = 1.6 Hz, 1H, 5-H), 7.05 (d, *^3^J_2″/N″_* = 2.4 Hz, 1H, 2″-H), 4.70 (t, *^3^J_4/3,3_* = 5.1 Hz, 1H, 4-H), 4.67 (t, *^3^J_1/2,2_* = 5.2 Hz, 1H, 1-H), 2.28–2.14 (m, 2H, 3,3-H), 2.05–1.91 (m, 2H, 2,2-H); *m*/*z* (ESI) 475.26 (100, [M−H]^−^).

(1*S*,4*S*)-1,4-Di(1*H*-indol-3-yl)-2,3,4,9-tetrahydro-1*H*-carbazole (**4a**). Yield 19%, white powder; mp 152–154 °C; ^1^H NMR (DMSO-d_6_) δ = 10.87 (d, *^3^J_N′/2′_* = 2,4 Hz, 1H, N′-H), 10.69 (d, *^3^J_N″/2″_* = 2,3 Hz, 1H, N″-H), 10.39 (s, 1H, N-H), 7.45 (d_br_, *^3^J_4″/5″_* = 7.9 Hz, *^4^J_4″/6″_*, 1H, 4″-H), 7.36 (d_br_, *^3^J_7′/6′_* = 8.1 Hz, *^4^J_7′/5′_*, 1H, 7′-H), 7.34 (d_br_, *^3^J_7″/6″_* = 8.1 Hz, *^4^J_7″/5″_*, 1H, 7″-H), 7.30 (d_br_, *^3^J_4′/5′_* = 7.9 Hz, *^4^J_4′/6′_*, 1H, 4′-H), 7.15 (d_br_, *^3^J_8/7_* = 8.1 Hz, *^4^J_8/6_*, 1H, 8-H), 7.05 (ddd, *^3^J_6′/7′_* = 8.1 Hz, *^3^J_6′/5′_* = 6.9 Hz, *^4^J_6′/4′_* = 1.1 Hz, 1H, 6′-H), 7.03 (ddd, *^3^J_6″/7″_* = 8.1 Hz, *^3^J_6″/5″_* = 6.9 Hz, *^4^J_6″/4″_* = 1.0 Hz, 1H, 6″-H), 6.99 (d, *^3^J_2′/N′_* = 2.4 Hz, 1H, 2′-H), 6.89 (ddd, *^3^J_5″/4″_* = 7.9 Hz, *^3^J_5″/6″_* = 6.9 Hz, *^4^J_5″/7″_* = 1.0 Hz, 1H, 5″-H), 6.87 (ddd, *^3^J_5′/4′_* = 7.9 Hz, *^3^J_5′/6′_* = 6.9 Hz, *^4^J_5′/7′_* = 1.0 Hz, 1H, 5′-H), 6.85 (d, *^3^J_2″/N″_* = 2.3 Hz, 1H, 2″-H), 6.84 (ddd, *^3^J_7/8_* = 8.1 Hz, *^3^J_7/6_* = 7.0 Hz, *^4^J_7/5_* = 1.0 Hz, 1H, 7-H), 6.81 (d_br_, *^3^J_5/6_* = 8.0 Hz, *^4^J_5/7_*, 1H, 5-H), 6.63 (ddd, *^3^J_6/5_* = 8.0 Hz, *^3^J_6/7_* = 7.0 Hz, *^4^J_6/8_* = 1.0 Hz, 1H, 6-H), 4.59 (t, *^3^J_1/2,2_* = 4.9 Hz, 1H, 1-H), 4.59 (t, *^3^J_4/3,3_* = 4.9 Hz, 1H, 4-H), 2.27–2.20 (m, 2H, 3,3-H), 2.03–1.96 (m, 2H, 2,2-H); *m*/*z* (ESI) 400.31 (100, [M−H]^−^); 801.24 (42, [2M−H]^−^).

(1*S*,4*S*)-6-Chloro-1,4-bis(5-chloro-1*H*-indol-3-yl)-2,3,4,9-tetrahydro-1*H*-carbazole (**4b**). Yield 12%, beige powder; mp 167–170 °C; ^1^H NMR (DMSO-d_6_) δ = 11.13 (d, *^3^J_N′/2′_* = 2.3 Hz, 1H, N′-H), 10.97 (d, *^3^J_N″/2″_* = 2.3 Hz, 1H, N″-H), 10.65 (s, 1H, N-H), 7.39 (d, *^3^J_7′/6′_* = 8.5 Hz, 1H, 7′-H), 7.38 (d, *^4^J_4″/6″_* = 2.0 Hz, 1H, 4″-H), 7.36 (d, *^3^J_7″/6″_* = 8.5 Hz, 1H, 7″-H), 7.29 (d, *^4^J_4′/6′_* = 2.0 Hz, 1H, 4′-H), 7.16 (d, *^3^J_8/7_* = 8.5 Hz, 1H, 8-H), 7.13 (d, *^3^J_2′/N′_* = 2.3 Hz, 1H, 2′-H), 7.05 (dd, *^3^J_6′/7′_* = 8.5 Hz, *^4^J_6′/4′_* = 2.0 Hz, 1H, 6′-H), 7.04 (d, *^3^J_2″/N″_* = 2.3 Hz, 1H, 2″-H), 7.02 (dd, *^3^J_6″/7″_* = 8.5 Hz, *^4^J_6″/4_* = 2.0 Hz, 1H, 6″-H), 6.85 (dd, *^3^J_7/8_* = 8.5 Hz, *^4^J_7/5_* = 2.1 Hz, 1H, 7-H), 6.70 (d, *^4^J_5/7_* = 2.1 Hz, 1H, 5-H), 4.64–4.60 (m, 1H, 1-H), 4.58–4.54 (m, 1H, 4-H), 2.23–2.17 (m, 2H, 3,3-H), 2.00–1.93 (m, 2H, 2,2-H); *m*/*z* (ESI) 502.38 (100, [M−H]^−^); 504.32 (92, [M−H]^−^).

(1*S*,4*S*)-7-Chloro-1,4-bis(6-chloro-1*H*-indol-3-yl)-2,3,4,9-tetrahydro-1*H*-carbazole (**4c**). Yield 13%, beige powder; mp 159–161 °C; ^1^H NMR (DMSO-d_6_) δ = 11.07 (d, *^3^J_N′/2′_* = 2.4 Hz, 1H, N′-H), 10.88 (d, *^3^J_N″/2″_* = 2.4 Hz, 1H, N″-H), 10.70 (s, 1H, N-H), 7.57 (d, *^3^J_4″/5″_* = 8.5 Hz, 1H, 4″-H), 7.42 (d, *^4^J_7′/5′_* = 1.9 Hz, 1H, 7′-H), 7.39 (d, *^3^J_4′/5′_* = 8.5 Hz, 1H, 4′-H), 7.39 (d, *^4^J_7″/5″_* = 1.9 Hz, 1H, 7″-H), 7.21 (d, *^4^J_8/6_* = 1.9 Hz, 1H, 8-H), 7.14 (d, *^3^J_2′/N′_* = 2.4 Hz, 1H, 2′-H), 6.96 (dd, *^3^J_5″/4″_* = 8.5 Hz, *^4^J_5″/7″_* = 1.9 Hz, 1H, 5″-H), 6.95 (dd, *^3^J_5′/4′_* = 8.5 Hz, *^4^J_5′/7′_* = 1.9 Hz, 1H, 5′-H), 6.89 (d, *^3^J_2″/N″_* = 2.4 Hz, 1H, 2″-H), 6.84 (d, *^3^J_5/6_* = 8.4 Hz, 1H, 5-H), 6.75 (dd, *^3^J_6/5_* = 8.4 Hz, *^3^J_6/8_* = 1.9 Hz, 1H, 6-H), 4.55–4.47 (m, 2H: 1- H; 4-H), 2.25–2.17 (m, 2H, 3,3-H), 2.04–1.97 (m, 2H, 2,2-H); *m*/*z* (ESI) 502.28 (100, [M−H]^−^); 504.31 (84, [M−H]^−^).

(1*S*,4*S*)-6-Bromo-1,4-bis(5-bromo-1*H*-indol-3-yl)-2,3,4,9-tetrahydro-1*H*-carbazole (**4d**). Yield 17%, beige powder; mp 115–117 °C; ^1^H NMR (DMSO-d_6_) δ = 11.15 (d, *^3^J_N′/2′_* = 2.3 Hz, 1H, N′-H), 11.00 (d, *^3^J_N″/2″_* = 2.4 Hz, 1H, N″-H), 10.69 (s, 1H, N-H), 7.57 (d, *^4^J_4″/6″_* = 1.9 Hz, 1H, 4″-H), 7.47 (d, *^4^J_4′/6′_* = 1.9 Hz, 1H, 4′-H), 7.36 (d, *^3^J_7′/6′_* = 8.6 Hz, 1H, 7′-H), 7.34 (d, *^3^J_7″/6″_* = 8.6 Hz, 1H, 7″-H), 7.18 (dd, *^3^J_6′/7′_* = 8.6 Hz, *^4^J_6′/4′_* = 1.9 Hz, 1H, 6′-H), 7.15 (dd, *^3^J_6″/7″_* = 8.6 Hz, *^4^J_6″/4″_* = 1.9 Hz, 1H, 6″-H), 7.15 (d, *^3^J_8/7_* = 8.5 Hz, 1H, 8-H), 7.10 (d, ^3^*J_2′/N′_* = 2.3 Hz, 1H, 2′-H), 7.00 (d, ^3^*J_2″/N″_* = 2.4 Hz, 1H, 2″-H), 6.99 (dd, *^3^J_7/8_* = 8.5 Hz, *^4^J_7/5_* = 2.0 Hz, 1H, 7-H), 6.89 (d, *^4^J_5/7_* = 2.0 Hz, 1H, 5-H), 4.63 (t, *^3^J_1/2,2_* = 4.9 Hz, 1H, 1-H), 4.58 (t, *^3^J_4/3,3_* = 4.9 Hz, 1H, 4-H), 2.25–2.18 (m, 2H, 3,3-H), 2.00–1.94 (m, 2H, 2,2-H); *m*/*z* (ESI) 636.18 (100, [M−H]^−^); 638.13 (91, [M−H]^−^); 634.22 (41, [M−H]^−^); 640.13 (32, [M−H]^−^).

(1*S*,4*S*)-7-Bromo-1,4-bis(5-bromo-1*H*-indol-3-yl)-2,3,4,9-tetrahydro-1*H*-carbazole (**4e**). Yield 12%, light pink powder; mp 149–152 °C; ^1^H NMR (DMSO-d_6_) δ = Hz, 1H, N′-H), 10.91 (d, *^3^J_N″/2″_* = 2.3 Hz, 1H, N″-H), 10.57 (s, 1H, N-H), 7.57 (d, *^4^J_7′/5′_* = 1.8 Hz, 1H, 7′-H), 7.52 (d, *^4^J_7″/5″_* = 1.8 Hz, 1H, 7″-H), 7.30 (d, *^4^J_8/6_* = 1.8 Hz, 1H, 8-H), 7.28 (d, *^3^J_4″/5″_* = 8.4 Hz, 1H, 4″-H), 7.19 (d, *^3^J_4′/5′_* = 8.4 Hz, 1H, 4′-H), 7.13 (d, *^3^J_2′/N′_* = 2.3 Hz, 1H, 2′-H), 7.03 (dd, *^3^J_5′/4′_* = 8.4 Hz, *^4^J_5′/7′_* = 1.8 Hz, 1H, 5′-H), 7.01 (d, *^3^J_2″/N″_* = 2.3 Hz, 1H, 2″-H), 6.98 (dd, *^3^J_5″/4″_* = 8.4 Hz, *^4^J_5″/7″_* = 1.8 Hz, 1H, 5″-H), 6.78 (dd, *^3^J_6/5_* = 8.4 Hz, *^4^J_6/8_* = 1.8 Hz, 1H, 6-H), 6.65 (d, *^3^J_5/6_* = 8.4 Hz, 1H, 5-H), 4.61–4.54 (m, 2H, 1-H; 4-H), 2.24–2.19 (m, 2H, 3,3-H), 2.03–1.97 (m, 2H, 2,2-H); *m*/*z* (ESI) 638.11 (100, [M−H]^−^); 636.13 (83, [M−H]^−^). 

(1*S*,4*S*)-6-(Benzyloxy)-1,4-bis(5-(benzyloxy)-1*H*-indol-3-yl)-2,3,4,9-tetrahydro-1*H*-carbazole (**4f**). Yield 20%, brownish powder; mp 95–96 °C; ^1^H NMR (DMSO-d_6_) δ = 10.71 (d, *^3^J_N′/2′_* = 2.3 Hz, 1H, N′-H), 10.56 (d, *^3^J_N″/2″_* = 2.4 Hz, 1H, N″-H), 10.26 (s, 1H, N-H), 7.40 (dd, *^3^J_2,6-Ph′/3,5-Ph′_* = 7.9 Hz, *^4^J_2,6-Ph′/4-Ph′_* = 1.1 Hz, 2H, 2,6-Ph′-H), 7.40–7.20 (m, 13H, Ar-H), 7.25 (d, *^3^J_7″/6″_* = 8.6 Hz, 1H, 7″-H), 7.24 (d, *^3^J_7′/6′_* = 8.6 Hz, 1H, 7′-H), 7.08 (d, *^3^J_8/7_* = 8.6 Hz, 1H, 8-H), 7.06 (d, *^4^J_4′/6′_* = 2.3 Hz, 1H, 4′-H), 6.94 (d, *^3^J_2′/N′_* = 2.3 Hz, 1H, 2′-H), 6.81 (d, *^3^J_2″/N″_* = 2.4 Hz, 1H, 2″-H), 6.79 (dd, *^3^J_6″/7″_* = 8.6 Hz, *^4^J_6″/4″_* = 2.4 Hz, 1H, 6″-H), 6.77 (dd, *^3^J_6′/7′_* = 8.6 Hz, *^4^J_6′/4′_* = 2.3 Hz, 1H, 6′-H), 6.75 (d, *^4^J_4″/6″_* = 2.4 Hz, 1H, 4″-H), 6.61 (dd, *^3^J_7/8_* = 8.6 Hz, *^4^J_7/5_* = 2.5 Hz, 1H, 7-H), 6.47 (d, *^4^J_5/7_* = 2.5 Hz, 1H, 5-H), 5.01 (s, 2H, O-CH_2_-Ph′), 4.84 (s, 2H, O-CH_2_-Ph″), 4.76–4.66 (m, 2H, O-CH_2_-Ph), 4.52–4.45 (m, 2H, 1-H; 4-H), 2.20–2.10 (m, 2H, 3,3-H), 2.01–1.81 (m, 2H, 2,2-H); *m*/*z* (ESI) 742.33 (100, [M+Na]^+^); 720.26 (13, [M+H]^+^).

(1*S*,4*S*)-7-(Benzyloxy)-1,4-bis(5-(benzyloxy)-1*H*-indol-3-yl)-2,3,4,9-tetrahydro-1*H*-carbazole (**4g**). Yield 25%, light brownish powder; mp 92–93 °C; ^1^H NMR (DMSO-d_6_) δ = 10.66 (d, *^3^J_N′/2′_* = 2.3 Hz, 1H, N′-H), 10.50 (d, *^3^J_N″/2″_* = 2.3 Hz, 1H, N″-H), 10.15 (s, 1H, N-H), 7.50–7.26 (m, 15H, Ar-H), 7.27 (d, *^3^J_4″/5″_* = 8.8 Hz, 1H, 4″-H), 7.14 (d, *^3^J_4′/5′_* = 8.8 Hz, 1H, 4′-H), 6.94 (d, *^4^J_7′/5′_* = 2.3 Hz, 1H, 7′-H), 6.91 (d, *^4^J_7″/5″_* = 2.3 Hz, 1H, 7″-H), 6.88 (d, *^3^J_2′/N′_* = 2.3 Hz, 1H, 2′-H), 6.76 (d, *^3^J_2″/N″_* = 2.3 Hz, 1H, 2″-H), 6.76 (d, *^4^J_8/6_* = 2.3 Hz, 1H, 8-H), 6.68 (d, *^3^J_5/6_* = 8.6 Hz, 1H, 5-H), 6.64 (dd, *^3^J_5′/4′_* = 8.8 Hz, *^4^J_5′/7′_* = 2.3 Hz, 1H, 5′-H), 6.62 (dd, *^3^J_5″/4″_* = 8.8 Hz, *^4^J_5″/7″_* = 2.3 Hz, 1H, 5″-H), 6.41 (dd, *^3^J_6/5_* = 8.6 Hz, *^4^J_6/8_* = 2.3 Hz, 1H, 6-H), 5.10 (s, 2H, O-CH_2_-Ph′), 5.09 (s, 2H, O-CH_2_-Ph″), 4.98 (s, 2H, O-CH_2_-Ph), 4.47 (t, *^3^J_1/2,2; 4/3,3_* = 5.9 Hz, 2H, 1-H; 4-H), 2.21–2.15 (m, 2H, 3,3-H), 1.99–1.93 (m, 2H, 2,2-H); *m*/*z* (ESI) 742.22 (100, [M+Na]^+^).

3,3′-((1*S*,4*S*)-6-Cyano-2,3,4,9-tetrahydro-1*H*-carbazole-1,4-diyl)bis(1*H*-indole-5-carbonitrile) (**4h**). Yield 9%, white powder; mp 182–184 °C; ^1^H NMR (DMSO-d_6_) δ = 11.58 (d, *^3^J_N′/2_* = 2.3 Hz, 1H, N′-H), 11.44 (d, *^3^J_N″/2″_* = 2.4 Hz, 1H, N″-H), 11.15 (s, 1H, N-H), 7.90 (d, *^4^J_4″/6″_* = 1.5 Hz, 1H, 4″-H), 7.87 (d, ^4^*J_4′/6′_* = 1.5 Hz, 1H, 4′-H), 7.57 (d, *^3^J_7′/6′_* = 8.5 Hz, 1H, 7′-H), 7.55 (d, *^3^J_7″/6″_* = 8.6 Hz, 1H, 7″-H), 7.43 (dd, *^3^J_6′/7′_* = 8.5 Hz, *^4^J_6′/4′_* = 1.5 Hz, 1H, 6′-H), 7.40 (dd, *^3^J_6″/7″_* = 8.6 Hz, *^4^J_6″/4″_* = 1.5 Hz, 1H, 6″-H), 7.34 (d, *^3^J_8/7_* = 8.4 Hz, 1H, 8-H), 7.28 (d, *^3^J_2′/N′_* = 2.3 Hz, 1H, 2′-H), 7.25 (dd, *^3^J_7/8_* = 8.4 Hz, *^4^J_7/5_* = 1.6 Hz, 1H, 7-H), 7.21 (d, *^3^J_2″/N″_* = 2.4 Hz, 1H, 2″-H), 7.12 (d, *^4^J_5/7_* = 1.6 Hz, 1H, 5-H), 4.79 (t, *^3^J_1/2,2_* = 5.2 Hz, 1H, 1-H), 4.74 (t, *^3^J_4/3,3_* = 5.2 Hz, 1H, 4-H), 2.29–2.23 (m, 2H, 3,3-H), 2.06–2.00 (m, 2H, 2,2-H); *m*/*z* (ESI) 475.23 (100, [M−H]^−^).

### 3.3. General Procedure for the Synthesis of Bisindolyl Carbazoles ***5***

An equimolar mixture of each compound **3** and **4** with the same indole substituent was dissolved in methanol (25 mL) and one and a half molar amount of 2,3-dichloro-5,6-dicyano-*p*-benzoquinone (DDQ) was added. The mixture was stirred at room temperature for 3 h protected from light. The reaction proceeding was followed by TLC and when no more detectable product formation was observed, the solvent was removed in vacuum. The remaining mixture was separated by column chromatography over silica gel. The eluated compound fractions were unified and the solvent was removed in vacuum to yield the solid compounds **5**.

1,4-Di(1*H*-indol-3-yl)-9*H*-carbazole (**5a**). Yield 59%, light beige powder; mp 296–271 °C; ^1^H NMR (DMSO-d_6_) δ = 11.52 (d, *^4^J_N′/2′_* = 2.5 Hz, 1H, N′-H), 11.38 (d, *^4^J_N″/2″_* = 2.5 Hz, 1H, N″-H), 10.96 (s, 1H, N-H), 7.84 (d, *^4^J_2′/N′_* = 2.5 Hz, 1H, 2′-H), 7.75 (d_br_, *^3^J_4′/5′_* = 8.0 Hz,*^4^J_4′/6′_,* 1H, 4′-H), 7.62 (d, *^4^J_2″/N″_* = 2.5 Hz, 1H, 2″-H), 7.61 (d, *^3^J_2/3_* = 7.5 Hz, 1H, 2-H), 7.55 (d_br_, *^3^J_7′/6′; 7″/6″_* = 8.3 Hz,*^4^J_7′/5′; 7″/5″_,* 2H, 7′-H; 7″-H), 7.53 (d_br_, *^3^J_8/7_* = 8.3 Hz,*^4^J_8/6_*, 1H, 8-H), 7.30 (d_br_, *^3^J_5/6_* = 8.1 Hz, *^4^J_5/7_,* 1H, 5-H), 7.29 (d_br_, *^3^J_4″/5″_* = 8.0 Hz,*^4^J_4″/6″_,* 1H, 4″-H), 7.24 (ddd, *^3^J_7/8_* = 8.3 Hz, *^3^J_7/6_* = 7.1 Hz, *^4^J_7/5_* = 1.2 Hz, 1H, 7-H), 7.22 (d, *^3^J_3/2_* = 7.5 Hz, 1H, 3-H), 7.21 (ddd, *^3^J_6′/7′_* = 8.3 Hz, ^3^*J_6′/5′_* =7.0 Hz, *^4^J_6′/4′_* =1.2 Hz, 1H, 6′-H), 7.18 (ddd, *^3^J_6″/7″_* = 8.3 Hz, *^3^J_6″/5″_* =6.9 Hz, *^4^J_6″/4″_* =1.2 Hz, 1H, 6″-H), 7.11 (ddd, *^3^J_5′/4′_* = 8.0 Hz, *^3^J_5′/6′_* = 7.0 Hz, *^4^J_5′/7′_* = 1.1 Hz, 1H, 5′-H), 6.97 (ddd, *^3^J_5″/4″_* = 8.0 Hz, *^3^J_5″/6″_* = 6.9 Hz, *^4^J_5″/7″_* = 1.1 Hz, 1H, 5″-H), 6.79 (ddd, *^3^J_6/5_* = 8.1 Hz, *^3^J_6/7_* = 7.1 Hz, *^4^J_6/8_* = 1.1 Hz, 1H, 6-H); *m*/*z* (ESI) 817.05 (100, [2M + Na]^+^); 420.26 (52, [M+Na]^+^).

6-Chloro-1,4-bis(5-chloro-1*H*-indol-3-yl)-9*H*-carbazole (**5b**). Yield 50%, yellow green powder; mp 134–137 °C; ^1^H NMR (DMSO-d_6_) δ = 11.75 (d, *^3^J_N′/2′_* = 2.5 Hz, 1H, N′-H), 11.67 (d, *^3^J_N″/2″_* = 2.5 Hz, 1H, N″-H), 11.22 (s, 1H, N-H), 7.91 (d, *^3^J_2′/N′_* = 2.5 Hz, 1H, 2′-H), 7.77 (d, *^3^J_2″/N″_* = 2.5 Hz, 1H, 2′-H), 7.66 (d, *^4^J_4′/6′_* = 2.1 Hz, 1H, 4′-H), 7.62 (d, *^3^J_2/3_* = 7.5 Hz, 1H, 2-H), 7.60 (d, *^3^J_7″/6″_* = 8.6 Hz, 1H, 7″-H), 7.57 (d, *^3^J_7′/6′_* = 8.6 Hz, 1H, 7′-H), 7.54 (d, *^3^J_8/7_* = 8.6 Hz, 1H, 8-H), 7.30 (dd, *^3^J_7/8_* = 8.6 Hz, *^4^J_7/5_* = 2.1 Hz, 1H, 7-H), 7.26 (d, *^3^J_3/2_* = 7.5 Hz, 1H, 3-H), 7.25 (d, *^4^J_4″/6″_* = 2.1 Hz, 1H, 4″-H), 7.22 (d, *^4^J_5/7_* = 2.1 Hz, 1H, 5-H), 7.22 (dd, *^3^J_6″/7″_* = 8.6 Hz, *^4^J_6″/4″_* = 2.1 Hz, 1H, 6″-H), 7.20 (dd, *^3^J_6′/7′_* = 8.6 Hz, *^4^J_6′/4′_* = 2.1 Hz, 1H, 6′-H); *m*/*z* (ESI) 498.45 (100, [M−H]^−^); 500.43 (95, [M−H]^−^); 502.44 (35, [M−H]^−^).

7-Chloro-1,4-bis(5-chloro-1*H*-indol-3-yl)-9*H*-carbazole (**5c**). Yield 26%, yellow powder; mp 168–170 °C; ^1^H NMR (DMSO-d_6_) δ = 11.68 (d, *^3^J_N′/2′_* = 2.3 Hz, 1H, N′-H), 11.56 (d, *^3^J_N″/2″_* = 2.3 Hz, 1H, N″-H), 11.13 (s, 1H, N-H), 7.86 (d, *^3^J_2′/N′_* = 2.3 Hz, 1H, 2′-H), 7.69 (d, *^3^J_2″/N″_* = 2.3 Hz, 1H, 2″-H), 7.68 (d, *^3^J_4′/5′_* = 8.2 Hz, 1H, 4′-H), 7.59 (d, *^3^J_2/3_* = 7.5 Hz, 1H, 2-H), 7.59 (d, *^4^J_7″/5″_* = 1.8 Hz, 1H, 7″-H), 7.59 (d, *^4^J_7′/5′_* = 1.9 Hz, 1H, 7′-H), 7.51 (d, *^4^J_8/7_* = 1.9 Hz, 1H, 8-H), 7.23 (d, *^3^J_3/2_* = 7.5 Hz, 1H, 3-H), 7.21 (d, *^3^J_4″/5″_* = 8.6 Hz, 1H, 4″-H), 7.18 (d, *^3^J_5/6_* = 8.5 Hz, 1H, 5-H), 7.12 (dd, *^3^J_5′/4′_* = 8.2 Hz, *^4^J_5′/7′_* = 1.9 Hz, 1H, 5′-H), 6.99 (dd, *^3^J_5″/4″_* = 8.6 Hz, *^4^J_5″/7″_* = 1.8 Hz, 1H, 5″-H), 6.86 (dd, *^3^J_6/5_* = 8.5 Hz, *^4^J_6/8_* = 1.9 Hz, 1H, 6-H); *m*/*z* (ESI) 498.54 (100, [M−H]^−^).

6-Bromo-1,4-bis(5-chloro-1*H*-indol-3-yl)-9*H*-carbazole (**5d**). Yield 51%, yellow green powder; mp 101–105 °C; ^1^H NMR (DMSO-d_6_) δ = 11.76 (d, *^3^J_N′/2′_* = 2.5 Hz, 1H, N′-H), 11.69 (d, ^3^*J_N″/2″_* = 2.5 Hz, 1H, N″-H), 11.23 (s, 1H, N-H), 7.89 (d, *^3^J_2′/N′_* = 2.5 Hz, 1H, 2′-H), 7.80 (d, *^4^J_4′/6′_* = 1.9 Hz, 1H, 4′-H), 7.75 (d, *^3^J_2″/N″_* = 2.5 Hz, 1H, 2″-H), 7.62 (d, *^3^J_2/3_* = 7.5 Hz, 1H, 2-H), 7.55 (d, *^3^J_7′/6′_* = 8.5 Hz, 1H, 7′-H), 7.53 (d, *^3^J_7″/6″_* = 8.6 Hz, 1H, 7″-H), 7.50 (d, *^3^J_8/7_* = 8.5 Hz, 1H, 8-H), 7.41 (dd, *^3^J_7/8_* = 8.5 Hz, *^4^J_7/5_* = 2.0 Hz, 1H, 7-H), 7.39 (d, *^4^J_4″/6″_* = 2.0 Hz, 1H, 4″-H), 7.38 (d, *^4^J_5/7_* = 2.0 Hz, 1H, 5-H), 7.33 (dd, *^3^J_6′/7′_* = 8.5 Hz, *^4^J_6′/4′_* = 1.9 Hz, 1H, 6′-H), 7.32 (dd, *^3^J_6″/7″_* = 8.6 Hz, *^4^J_6″/4″_* = 2.0 Hz, 1H, 6″-H), 7.26 (d, *^3^J_3/2_* = 7.5 Hz, 1H, 3-H); *m*/*z* (ESI) 632.32 (100, [M−H]^−^); 634.27 (98, [M−H]^−^); 636.26 (36, [M−H]^−^); 630.31 (31, [M−H]^−^).

7-Bromo-1,4-bis(5-chloro-1*H*-indol-3-yl)-9*H*-carbazole (**5e**). Yield 26%, beige powder; mp 180–183 °C; ^1^H NMR (DMSO-d_6_) δ = 11.69 (d, *^3^J_N′/2′_* = 2.5 Hz, 1H, N′-H), 11.57 (d, *^3^J_N′/2′_* = 2.5 Hz, 1H, N′-H), 10.13 (s, 1H, N-H), 7.85 (d, *^3^J_2′/N′_* = 2.5 Hz, 1H, 2′-H), 7.74 (d, *^4^J_7′/5′_* = 1.8 Hz, 1H, 7′-H), 7.73 (d, *^4^J_7″/5″_* = 1.8 Hz, 1H, 7″-H), 7.68 (d, *^3^J_2″/N″_* = 2.5 Hz, 1H, 2″-H), 7.67 (d, *^4^J_8/6_* = 1.9 Hz, 1H, 8-H), 7.63 (d, *^3^J_4′/5′_* = 8.5 Hz, 1H, 4′-H), 7.60 (d, *^3^J_2/3_* = 7.5 Hz, 1H, 2-H), 7.24 (dd, *^3^J_5′/4′_* = 8.5 Hz, *^4^J_5′/7′_* = 1.8 Hz, 1H, 5′-H), 7.23 (d, *^3^J_3/2_* = 7.5 Hz, 1H, 3-H), 7.16 (d, *^3^J_4″/5″_* = 8.5 Hz, 1H, 4″-H), 7.13 (d, *^3^J_5/6_* = 8.7 Hz, 1H, 5-H), 7.10 (dd, *^3^J_5″/4″_* = 8.5 Hz, *^4^J_5″/7″_* = 1.8 Hz, 1H, 5″-H), 6.99 (dd, *^3^J_6/5_* = 8.7 Hz, *^4^J_6/8_* = 1.9 Hz, 1H, 6-H); *m*/*z* (ESI) 634.35 (100, [M−H]^−^); 634.47 (93, [M−H]^−^); 636.31 (42, [M−H]^−^); 630.57 (26, [M−H]^−^); 636.32 (12, [M−H]^−^).

6-(Benzyloxy)-1,4-Bis(5-(benzyloxy)-1*H*-indol-3-yl)-9*H*-carbazole (**5f**). Yield 24%, yellow green powder; mp 109–112 °C; ^1^H NMR (DMSO-d_6_) δ = 11.36 (d, *^3^J_N′/2′_* = 2.5 Hz, 1H, N′-H), 11.30 (d, *^3^J_N″/2″_* = 2.5 Hz, 1H, N″-H), 10.75 (s, 1H, N-H), 7.77 (d, *^3^J_2′/N′_* = 2.5 Hz, 1H, 2′-H), 7.57 (d, *^3^J_2″/N″_* = 2.5 Hz, 1H, 2″-H), 7.51 (d, *^3^J_2/3_* = 7.5 Hz, 1H, 2-H), 7.51 (d, *^3^J_7″/6″_* = 8.7 Hz, 1H, 7″-H), 7.45 (dd, *^3^J_2,6-Ph′/3,5-Ph′_* = 8.4 Hz, *^4^J_2,6-Ph′/4-Ph′_* = 1.4 Hz, 2H, 2,6-Ph′-H), 7.44 (d, *^3^J_7′/6′_* = 8.5 Hz, 1H, 7′-H), 7.43 (d, *^3^J_8/7_* = 8.7 Hz, 1H, 8-H), 7.36 (ddd, *^3^J_3,5-Ph′/2,6-Ph′_* = 8.4 Hz, *^3^J_3,5-Ph′/4-Ph′_* = 6.8 Hz, *^4^J_3,5-Ph′/3,5-Ph′_* = 1.4 Hz, 2H, 3,5-Ph′-H), 7.30 (d, *^4^J_4′/6′_* = 2.4 Hz, 1H, 4′-H), 7.30 (dd, *^3^J_2,6-Ph″/3,5-Ph″_* = 8.5 Hz, *^4^J_2,6-Ph″/4-Ph″_* = 1.4 Hz, 2H, 2,6-Ph″-H), 7.34–7.20 (m, 7H, Ar-H), 7.16 (d, *^3^J_3/2_* = 7.5 Hz, 1H, 3-H), 7.12 (dd, *^3^J_2,6-Ph/3,5-Ph_* = 7.9 Hz, *^4^J_2,6-Ph/4-Ph_* = 1.4 Hz, 2H, 2,6-Ph-H), 6.98 (dd, *^3^J_6′/7′_* = 8.5 Hz, *^4^J_6′/4′_* = 2.4 Hz, 1H, 6′-H), 6.96 (dd, *^3^J_6″/7″_* = 8.7 Hz, *^4^J_6″/4″_* = 2.5 Hz, 1H, 6″-H), 6.96 (d, *^4^J_5/7_* = 2.5 Hz, 1H, 5-H), 6.94 (dd, *^3^J_7/8_* = 8.7 Hz, *^4^J_7/5_* = 2.5 Hz, 1H, 7-H), 6.89 (d, *^4^J_4″/6″_* = 2.5 Hz, 1H, 4″-H), 5.09 (s, 2H, O-CH_2_-Ph′), 4.89 (s, 2H, O-CH_2_-Ph″), 4.62 (s, 2H, O-CH_2_-Ph); *m*/*z* (ESI) 738.54 (100, [M+Na]^+^).

7-(Benzyloxy)-1,4-Bis(5-(benzyloxy)-1*H*-indol-3-yl)-9*H*-carbazole (**5g**). Yield 33%, light brownish powder; mp 112–115 °C; ^1^H NMR (DMSO-d_6_) δ = 11.28 (d, *^3^J_N′/2′_* = 2.5 Hz, 1H, N′-H), 11.14 (d, *^3^J_N″/2″_* = 2.5 Hz, 1H, N″-H), 10.79 (s, 1H, N-H), 7.65 (d, *^3^J_2′/N′_* = 2.5 Hz, 1H, 2′-H), 7.61 (d, *^3^J_4″/5″_* = 8.7 Hz, 1H, 4″-H), 7.51 (dd, *^3^J_2,6-Ph′/3,5-Ph′; 2,6-Ph″/3,5-Ph″_* = 8.0 Hz, *^4^J_2,6-Ph′/4-Ph′; 2,6-Ph″/4-Ph″_* = 1.3 Hz, 4H, 2,6-Ph′-H; 2,6-Ph″-H), 7.48 (d, *^3^J_2/3_* = 7.5 Hz, 1H, 2-H), 7.45 (dd, *^3^J_2,6-Ph/3,5-Ph_* = 8.0 Hz, *^4^J_2,6-Ph/4-Ph_* = 1.4 Hz, 2H, 2,6-Ph-H), 7.44 (d, *^3^J_2″/N″_* = 2.5 Hz, 1H, 2″-H), 7.42–7.31 (m, 9H, Ar-H), 7.20 (d, *^3^J_5/6_* = 8.7 Hz, 1H, 5-H), 7.14 (d, *^3^J_3/2_* = 7.5 Hz, 1H, 3-H), 7.14 (d, *^3^J_4′/5′_* = 8.7 Hz, 1H, 4′-H), 7.11 (d, *^4^J_8/6_* = 2.3 Hz, 1H, 8-H), 7.10 (d, *^4^J_7″/5″_* = 2.4 Hz, 1H, 7″-H), 7.09 (d, *^4^J_7′/5′_* = 2.4 Hz, 1H, 7′-H), 6.85 (dd, *^3^J_5″/4″_* = 8.7 Hz, *^4^J_5″/7″_* = 2.4 Hz, 1H, 5″-H), 6.71 (dd, *^3^J_5′/4′_* = 8.7 Hz, *^4^J_5′/7′_* = 2.4 Hz, 1H, 5′-H), 6.53 (dd, *^3^J_6/5_* = 8.7 Hz, *^4^J_6/8_* = 2.3 Hz, 1H, 6-H), 5.18 (s, 2H, O-CH_2_-Ph′), 5.16 (s, 2H, O-CH_2_-Ph″), 5.10 (s, 2H, O-CH_2_-Ph); *m*/*z* (ESI) 738.28 (100, [M+Na]^+^).

3,3′-(6-Cyano-9*H*-carbazole-1,4-diyl)bis(1*H*-indole-5-carbonitrile) (**5h**). Yield 29%, light pink powder; mp 190–193 °C; ^1^H NMR (DMSO-d_6_) δ = 12.16 (d, *^3^J_N′/2′_* = 2.5 Hz, 1H, N′-H), 12.12 (d, *^3^J_N″/2″_* = 2.5 Hz, 1H, N″-H), 11.71 (s, 1H, N-H), 8.16 (d, *^4^J_4′/6′_* = 1.6 Hz, 1H, 4′-H), 8.05 (d, *^3^J_2′/N′_* = 2.5 Hz, 1H, 2′-H), 8.00 (d, *^3^J_2″/N″_* = 2.5 Hz, 1H, 2″-H), 7.78 (d, *^3^J_7″/6″_* = 8.4 Hz, 1H, 7″-H), 7.76 (d, *^4^J_4″/6″_* = 1.6 Hz, 1H, 4″-H), 7.75 (d, *^3^J_2/3_* = 7.5 Hz, 1H, 2-H), 7.73 (d, *^3^J_7′/6′_* = 8.5 Hz, 1H, 7′-H), 7.69 (dd, *^3^J_7/8_* = 8.5 Hz, *^4^J_7/5_* = 1.5 Hz, 1H, 7-H), 7.68 (d, *^3^J_8/7_* = 8.5 Hz, 1H, 8-H), 7.58 (dd, *^3^J_6″/7″_* = 8.4 Hz, *^4^J_6″/4″_* = 1.6 Hz, 1H, 6″-H), 7.58 (dd, *^3^J_6′/7′_* = 8.4 Hz, *^4^J_6′/4′_* = 1.6 Hz, 1H, 6′-H), 7.55 (d, *^4^J_5/7_* = 1.5 Hz, 1H, 5-H), 7.39 (d, *^3^J_3/2_* = 7.5 Hz, 1H, 3-H); *m*/*z* (ESI) 471.53 (100, [M−H]^−^).

### 3.4. Determination of Antibacterial Activity

The compounds and the standards as positive control were dissolved in 12.5% dimethyl sulfoxide (DMSO) at concentrations of 128 µg/mL. The chosen DMSO concentration should avoid any precipitation during the following dilution procedures of the compounds which were easily dissolved in the solution. The 12.5% DMSO has been used as a negative control. Further dilutions of the compounds and used standard drugs oxacillin and ciprofloxacin in the test medium were prepared at the required quantities of 64, 32, 16, 8, 4, 2, 1, 0.5 and 0.25 µg/mL concentrations with Mueller-Hinton broth containing beef infusion solids (20 g/L), casein hydrolysate (17.5 g/L) and starch (1.5 g/L). The minimum inhibitory concentrations (MIC) were determined using the 2-fold serial dilution technique. All the compounds were tested for their in vitro growth inhibitory activity against MRSA USA300 LAC* lux with a bioluminescence gene [32] and in case of an activity <16 µg/mL, the respective compounds were tested against MRSA JE2 as a variant of USA300 LAC* with cured resistance genes located on the plasmids [33], against MSSA ATCC6538 and against the MSSA HG003. The cultures were obtained from Mueller-Hinton broth (Difco) for all the bacterial strains after 24 h of incubation at 37 ± 1 °C. Testing was carried out in Mueller-Hinton broth at pH 7.4. The final inoculum size was 5 × 10^5^ CFU/mL for the antibacterial assay. A set of tubes containing only inoculated broth was used as a control. After incubation for 24 h at 37 ± 1 °C, the last tube with no visible growth of microorganisms was recorded to represent the MIC (expressed in µg/mL). Thus, the procedure followed the recommendation of the Clinical and Laboratory Standards Institute guideline [34].

## 4. Conclusions

Due to the emerging resistance development to antibiotics of almost all classes, new compound classes and strategies are necessary to counteract and lower the resistance proceeding. It would be most favourable to find new compounds that are additionally available without enormous costs for production or synthesis to become attractive for pharmaceutical companies. Besides natural products with mostly more complicated structures, synthetic antibiotics are attractive, but their present number is limited. We followed the innovative strategy to find novel antibacterial agents by dimerizing indole compounds and an additional hybridization with carbazole derivatives. In this way, novel bisindole carbazoles and tetrahydrocarbazoles were available in a simple one-pot reaction with a final aromatizing reaction step. The best antibacterial activities towards MRSA and MSSA resulted for 5-chloro and -cyano indole and -hydrogen substitutions for the respective tetrahydrocarbazoles and carbazoles. Those tetrahydrocarbazoles with a hydrogen indole substitution exceed the MRSA activities of the used standard antibiotics and one perspective lead compound exceeds those activities by a factor of 32. This means a comparable outstanding activity. Moreover, our most active compounds show a better activity than recently published novel antibacterial agents against MRSA and *S. aureus* with activities ranging between 3 and 64 µg/mL [35]. Consequently, they are promising candidates for subsequent in vivo studies.

## Figures and Tables

**Table 1:** Antibacterial activity data of target compounds **3a**–**f**, **h**, **4a**–**h** and **5a**–**h** with varied substitution patterns expressed as MIC values.

Cpd.	MIC [µg/mL] ^[a]^					
	R^1^	R^2^	LAC*	JE2	ATCC6538	HG003
**3a**	H	H	1	2	1	2
**3b**	Cl	H	8	4	8	16
**3c**	H	Cl	16			
**3d**	Br	H	16			
**3e**	H	Br	>128			
**3f**	OBn	H	>128			
**3h**	CN	H	4	2	4	8
**4a**	H	H	4	16	2	32
**4b**	Cl	H	8	4	8	32
**4c**	H	Cl	16			
**4d**	Br	H	16			
**4e**	H	Br	>128			
**4f**	OBn	H	128			
**4g**	H	OBn	>128			
**4h**	CN	H	8	4	2	16
**5a**	H	H	8	4	4	4
**5b**	Cl	H	8	8	4	16
**5c**	H	Cl	16			
**5d**	Br	H	64			
**5e**	H	Br	>128			
**5f**	OBn	H	128			
**5g**	H	OBn	>128			
**5h**	CN	H	8	8	8	16
oxacillin			32	32	1	0.5
ciprofloxacin			32	32	2	1

^[a]^ Mean of three determinations.

## Data Availability

Data of the compounds are not available from the authors.

## Sample Availability

Samples of the compounds are not available from the authors.
